# The interplay between ADHD and school shift on educational outcomes in children and adolescents: A cross-sectional and longitudinal analysis

**DOI:** 10.21203/rs.3.rs-4364073/v1

**Published:** 2024-05-13

**Authors:** Ighor Miron Porto, João Villanova Amaral, João Pedro Gonçalves Pacheco, Igor Terra, Euripedes Constantino Miguel, Pedro Mario Pan, Ary Gadelha, Luis Augusto Rohde, Giovanni Abrahão Salum, Maurício Scopel Hoffmann

**Affiliations:** Universidade Federal de Santa Maria (UFSM); Universidade Federal do Rio Grande do Sul (UFRGS); Universidade Federal de Santa Maria (UFSM); Universidade Federal do Rio Grande do Sul (UFRGS); Universidade de São Paulo; Universidade Federal de São Paulo (UNIFESP); National Institute of Developmental Psychiatry for Children and Adolescents (INCT-CNPq); Universidade Federal do Rio Grande do Sul (UFRGS); Child Mind Institute; Universidade Federal de Santa Maria (UFSM)

**Keywords:** Attention-deficit and hyperactivity, sleep, reading, writing, educational achievement

## Abstract

Many countries implement a double-shift schooling system, offering morning or afternoon shifts, driven by diverse factors. Young people with ADHD may face educational problems attending morning shifts compared to afternoon shifts. To investigate this, we used data from a Brazilian school-based cohort (n = 2.240, 6–14 years old, 45.6% female; 50.2% in the morning shift; 11.2% with ADHD). ADHD was determined by child psychiatrists using semi-structured interview. Educational outcomes were measured cross-sectionally and three years later (80% retention) and included reading and writing ability, performance in school subjects, and any negative school events (repetition, suspension, or dropout). Generalized regression models tested the interaction between ADHD and school shift and were adjusted for age, sex, race/ethnicity, intelligence, parental education, socioeconomic status, and site. Attrition was adjusted with inverse probability weights. We used two dimensional measures of attentional problems as sensitivity analysis. ADHD and morning shift were independently associated with lower reading and writing ability and with higher odds for negative school events cross sectionally. ADHD independently predicted lower performance in school subjects and higher negative school events at follow-up. Interaction was found only at the cross-sectional level in a way that those studying in the afternoon present better educational outcomes compared with those studying in the morning only if they have lower ADHD symptom. Thus, ADHD was not associated with poorer educational outcomes among those studying in the morning. However, participants studying in the afternoon with lower levels of attentional problems presented better educational, despite these associations fade away over time.

## Introduction

Attention-deficit/hyperactivity disorder (ADHD) is a prevalent neurodevelopmental condition characterized by higher levels of inattention, and/or hyperactivity/impulsivity than developmentally expected, with pervasive impacts on academic performance and achievement during the life-course [1–3]. The impact of ADHD on education must be contextualized within children and adolescents’ circumstances. Globally, 1.6 billion children are enrolled in schools, with 1 billion in low- and middle-income countries. (LAMIC) [4]. Many schools in LAMIC use a double-shift system where students attend either morning, afternoon, or night shifts [5, 6]. This approach allows countries to optimize infrastructure, personnel, and other resources for education at the expense of quality [7]. In this scenario, children with ADHD can be assigned to either morning or afternoon shift and little is known about which is best for them.

Evidence suggests educational systems should accommodate the circadian preferences of children and adolescents with ADHD, who typically favour eveningness [8]. This natural inclination conflicts with early school start times, potentially worsening ADHD symptoms due to shortened sleep duration. Aligning chronotype and school schedule has shown to enhance academic performance for adolescents [10]. Studies indicate that delaying start times by one hour allows for longer sleep, resulting in improved academic performance, reduced tardiness, and absenteeism [11]. Recent research supports the benefits of timing adjustments on educational outcomes. In the USA, a delay of 50–65 minutes resulted in improved grade point averages, fewer late arrivals, and significantly reduced behaviour referrals [12]. Similarly, in Brazil, shifting from morning to afternoon classes enhanced Portuguese test scores for adolescents, while the reverse change decreased scores [13].

Despite evidence of an association between ADHD and educational problems, studies have yet to integrate these fifindings within the context of the double-shift schooling system, widely utilized in areas where most young people live. Therefore, it is relevant to investigate whether individuals with ADHD are particularly susceptible to poor educational outcomes when exposed to morning school shifts compared to those exposed exclusively to afternoon shifts. Furthermore, studies have not examined whether poor educational performance is transient (i.e., only associated cross-sectionally) or enduring (i.e., longitudinally associated).

To address these gaps, this study aims to investigate the hypothesis that children and adolescents with ADHD attending school in the morning may be at risk for poor educational outcomes when compared with those studying in the afternoon, and whether this association persists over time. We tested this by examining the interaction of ADHD and school shift in their cross-sectional and 3-year follow-up association with reading and writing ability, performance in school subjects and negative school events (suspension, repetition and dropout). Additionally, we aim to investigate whether this interaction occurs at both diagnostic and symptomatic levels, as previous studies primarily focused on levels of inattention symptoms rather than diagnosis. Drawing on existing literature, we hypothesize that ADHD and morning shift will have independent and interactive associations with poor educational outcomes, in a sense that those with ADHD will have poor educational outcomes if they study in the morning when compared with those studying in the afternoon. Furthermore, we hypothesize that this impact will be found cross-sectionally and longitudinally for both symptoms and diagnosis of ADHD. If confirmed, these findings could prompt a reassessment of educational policies and advocate for further research into the benefits of later school sessions for children and adolescents with ADHD.

## Methods

### Participants

We used baseline and first follow-up data from a large school-based community study – the Brazilian High-Risk Cohort Study [14]. At the beginning of the school year (2010), families were interviewed in 22 schools in Porto Alegre and 35 in São Paulo, two major cities in Brazil and capital of their states. 8,012 caregivers (87.3% mothers) accepted to be interviewed with a modified version of the Family History Screen [15] conducted by lay interviewers, with the purpose to generated a family liability index that expresses the percentage of family members who screened positive for a psychiatric condition [16]. Two subgroups were recruited from a total of 9,997 screened subjects: a random subsample (n = 957) and a high family risk sub-sample based on the family liability index (n = 1,554). Thus, a total of 2511 subjects, from 6 to 14 years of age, and their caregiver, were invited to participate at baseline, with a new invitation 3-year later (n = 2010 participants; 80% retention; 9–17 years of age, 44% female). Household parent report was collected by lay interviewers and youth assessment (self-reports and tests) was conducted by trained psychologists. The sample is representative of the economic social class in Brazil at baseline (A class = 0.5%; B class = 29.6%; C class = 64%; D and E class = 6.0%; A being the richest and E the poorest) [17]. Follow-up participation was associated with higher maternal education and socioeconomic status and living in Porto Alegre [18]. Weights were calculated to account for sampling procedure and attrition (see below).

### Predictors

School shift was informed by the caregivers and strati ed in three shifts: morning (n = 970), afternoon (n = 1116) and full day (n = 154). To this study, morning and full day were grouped together because students have morning routines in this category (n = 1124). In Brazil, parents do not choose school shifts; rather, shifts are assigned based on vacancy availability.

ADHD was assessed using the Development and Well-Being Assessment (DAWBA) [19], Brazilian Portuguese version [20]. This structured interview was administered to the caregivers by trained lay interviewers and scored by trained child psychiatrists who were supervised by a senior child psychiatrist [14]. As ADHD symptoms vary in a continuum in the population [21, 22], we also assessed symptoms of ADHD at baseline using two questionnaires, as a sensitivity analysis. For that, we used the Strengths and Difficulties Questionnaire (SDQ) and the Child Behavior Checklist (CBCL), reported by the caregiver about child and adolescents emotional and behavioural problems over the past six months [23, 24]. SDQ consisted in a 20-item questionnaire on behavioural and emotional difficulties plus five pro-social strengths. Responses were given as “Not true”, “Somewhat true” and “Certainly true” on how each attribute applied to the child or adolescent. For the purposes of this study, we scored each subject using confirmatory factor analysis (CFA) and included the four difficulties subscales: attention/hyperactivity (SDQ-hyper), emotional, conduct and peer problems [23]. CBCL is composed by 120 items on emotional and behavioural problems answered in a 3-point scale (0 = not true; 1 = somewhat/sometimes true; 2 = very true/often). We used a validated factor model including 66 items, composed by an attention/hyperactivity dimension (CBCL-att), plus internalising and externalising dimensions [25]. We used the SDQ-hyper and CBCL-att variables only for our analysis. Specific methods for factor analysis are described in supplementary information (page 1).

### School and Educational Outcomes

Reading and writing ability were measured throughout participants’ scores on the School Performance Test (“Teste de Desempenho Escolar” – TDE) [26]. The test is composed of right/wrong evaluations of 61 read decoding items and 12 writing items (writing words based on oral dictation) to assess its respective ability, at baseline and follow-up. A correlated two-factor model was previously estimated [27] and factor scores or reading and writing abilities at baseline and follow-up were extracted and used in the present analysis.

The performance in school subjects was measured using the CBCL, informed by the caregiver (CBCL-school) [28]. The CBCL contains eight items about how good the child/adolescent is in school subjects, compared with their peers. The school subjects are Portuguese or literature, history or social studies, English or Spanish, mathematics, biology, sciences, geography, and computer studies performance. Each subject was scored as 0 (failing), 1 (below average), 5 (average), and 10 (above average). We used factors scores from a longitudinal and unidimensional factor model previously described and validated in this sample [27].

Negative school events consisted of any caregiver’s report of school suspension, repetition, and dropout, and was coded as a binary variable (no/yes).

### Covariates

We adjusted the analyses for the following potentially confounding baseline variables: age (in years); socioeconomic group (SEG); race/ethnicity, categorized as white and non-white (black, mixed, Asian and indigenous participants); intelligence (IQ); educational level ( 1st, 2nd, 3rd, 4th, 5th, 6th, 7th, 8th and 9th year of primary education); parental highest education level (PEL) (No/incomplete primary, Complete primary, Complete secondary, Complete tertiary); and study site (due to differences in state-level legislation on retention and prevalence of psychiatric conditions). Details on these covariates are described in the supplementary information (page 3).

### Oversampling and inverse probability weights

The BHRCS used an oversampling procedure for high family risk for mental health problems, as described above. We then generated a propensity score weight to account for this high-risk stratification, which fully accounted for the oversampling procedure [29]. In previous analysis of the BHRCS, baseline maternal education, any child’s anxiety condition and study site predicted response at follow-up [18]. Therefore, we used these variables to compute inverse probability weight (IPW) to address sample attrition [30]. Sampling weight and IPW were multiplied and trimmed for the 10th and 90th percentile (final weights between 0.1337 and 3.8817). The resulting weights were used throughout the analyses to minimize bias associated with oversampling for high-risk procedures and missing data at follow-up, so the sample is closer to the general population at baseline.

### Statistical Analysis

We estimated linear (reading and writing abilities, and school achievement score) and logistic (negative school events) regression models, adjusted for the aforementioned covariates and weighted to account for missing data at follow-up (see below). All regression models contained interaction term between school shift and ADHD (diagnostic and dimensional levels) to evaluate if these variables moderate each other while associating with educational outcomes. Analyses were performed cross-sectionally and longitudinally. In longitudinal analysis we also included the baseline level of the educational outcome to estimate the independent effects of the predictors. As sensitivity analysis, we estimated additional models replacing ADHD for dimensional measures of inattention/hyperactivity problems using factor scores derived from SDQ and CBCL factor models. For full description of the statistical analysis and modelling of these factors, see supplementary information (page 1).

All the aforementioned linear and logistic regression models were weighted for high-risk sampling and for attrition as described above. All analysis were performed using R (version 4. 2.0 2022–04-22) in RStudio software version 2023.09.1 + 494 with the packages *lavaan* [31] for con rmatory factor analysis, *margins* [32] to estimate marginal effects and *interactions* [33] to graphically represent the interactions found.

## Results

### Factor models and sample description between baseline and follow‐up

Table 1 describes the sample’s characteristics at baseline stratified by school shift, with 1116 participants studying in the afternoon and 1124 in the morning (n=970) or full day (n=154), totalizing 2.240 students. School shifts were not different in terms of ADHD prevalence (or medication treatment for this condition), gender, participant’s race/ethnicity, IQ, parental education and social class (Table 1). Differences between school shifts were found in a way that participants in the morning shift were slightly older, are enrolled in higher educational level, are from São Paulo and presented lower levels of inattention/hyperactivity symptoms as measured by SDQ.

Factor models accounting for baseline psychopathology measured with SDQ and CBCL fitted the data well and generated reliable inattention/hyperactivity factors (see supplementary information page 2 for model results and Supplementary Tables A1 and A2 for details on SDQ and CBCL modelling and factor structure respectively).

Baseline and follow-up educational outcomes were stratified by school shift and ADHD (Table 2). At baseline and at 3-year follow-up, reading and writing abilities and performance in school subjects were lower for those ADHD in comparison with those without AHDH in both shifts. At baseline, repetition and suspension were higher for those with ADHD in the afternoon shift only. Those with ADHD and studying in the morning shift presented higher dropout/expulsion when compared with those without ADHD in the same shift at the follow-up.

### Relationship between ADHD, school shift and educational outcomes

**Figure 1:**
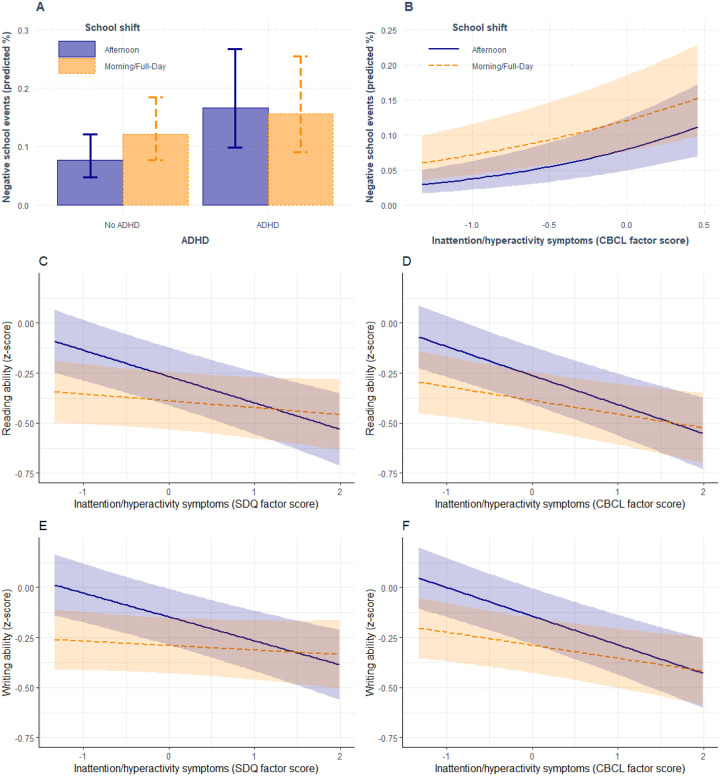
Interaction of school shift and attention-deficit and hyperactivity disorder (ADHD) or dimensional measures of ADHD (SDQ and CBCL) on the cross-sectional probability of educational outcomes Note: Negative school events are depicted in **A** for ADHD and **B** for CBCL. Reading is depicted in **C** for SDQ and **D** for CBCL, and writing abilities is depicted in **E** for SDQ and **F** for CBCL. Shades around solid (afternoon school shift) and dashed lines |(morning school shift) represent 95% confidence intervals. SDQ Strength and Difficulties Questionnaire; CBCL, Child and Behavioral Checklist.

Cross-sectional analysis demonstrated ADHD was associated with lower reading and writing abilities, lower performance in school subjects and higher chances of negative school events when compared with those without ADHD (Table 3). Morning school shift was associated with lower reading and writing ability, and higher negative school events when compared with afternoon shift (Table 3). Interactions between ADHD and school shift were also found. Those with ADHD in the morning shift presented lower performance in school subjects when compared with those in the afternoon shift (β = −0.33; 95% CI −0.58 – −0.09; p=0.008; Table 3 and Supplementary Figure A1) and those with ADHD in the morning shift presented lower negative school events when compared with those in the afternoon shift (OR = 0.56; 95% CI 0.31 – 0.98; p=0.044; Figure 1A and Table 3). However, only the later result was replicated when using dimensional measures of ADHD (Figure 1A and 1B). Graphical examination and marginal effect analysis demonstrates that, compared with those without ADHD, those with ADHD and studying in the afternoon shift present a probability for negative school events of 10.13% (95%CI 5.55 – 15.04%; p<0.001) while those in the morning shift presented a unsignificant difference of 3.64% (95%CI −1.83% – 9.12%; p=0.192) between those with and without ADHD for the probability of negative school events (Supplementary Table A3 and Figure 1A). This is because those studying in the morning already have a high probability for negative school events in the absence of ADHD (Figure 1A). Full cross-sectional analysis of ADHD and school shift with covariates is described in Supplementary Table A4.

Three years later, those with ADHD at baseline presented worse performance in school subjects and higher chance for negative school events, independently from covariates and baseline levels of these outcomes (Table 3 and Supplementary Table A5 for full results). These results were replicated with dimensional measures of ADHD (see below). No associations were found for baseline school shift, nor interactions with ADHD.

### Sensitivity analysis of ADHD symptoms, school shift and educational outcomes

Cross-sectional analysis using SDQ-hyper (i.e., SDQ inattention/hyperactivity factor score) replicated the main findings, described in Table 3, but not the interactions (Supplementary Table A6). In fact, SDQ-hyper presented significant interaction with school shift for reading and writing abilities. Students with higher SDQ-hyper and attending the morning shift had better reading (β = 0.10; 95% CI 0.03 – 0.17; p=0.006) and writing (β = 0.10 95% CI 0.03 – 0.16; p=0.005) for each one SDQ-hyper z-score increase relative to those studying in the afternoon (Figure 1C and 1D, Supplementary Table A6). Marginal effects demonstrate that those studying in the afternoon shift present a decreasing level of reading (β = −0.13; 95% CI −0.18 – −0.08; p<0.001) and writing ability (β = −0.12; 95% CI −0.17 – −0.07; p<0.001) for each one SDQ-hyper z-score increase, while those in the morning shift did not decrease their reading and writing abilities while increasing their inattention/hyperactivity symptoms (Supplementary Table A3). This is because reading and writing abilities are already low when compared with those in the afternoon shift (Figure 1C and 1D).

Three years later, higher SDQ-hyper at baseline was associated with worse performance in school subjects and higher chance for negative school events, independently from covariates and baseline levels of these outcomes, replicating the main findings (Supplementary Table A7). Morning shift at baseline also predicted higher chance for negative school events in the regression model using SDQ-based dimensional measure for inattention/hyperactivity (Supplementary Table A7).

Cross-sectional analysis using CBCL inattention/hyperactivity factor score (CBCL-att) replicated the main findings, described in Table 3, including the interaction for the negative school event outcome (Supplementary Table S8). Additionally, CBCL-att presented significant interaction with school shift for reading and writing abilities, replicating the findings with SDQ. Students with higher SDQ-hyper and attending the morning shift had better reading (β = 0.08; 95% CI 0.01 – 0.15; p=0.031) and writing (β = 0.08; 95% CI 0.01 – 0.15; p=0.022) for each one CBCL-att z-score increase relative to those studying in the afternoon (Figure 1E and 1F, Supplementary Table S8). Marginal effects demonstrate that those studying in the afternoon shift present a higher decreasing level of reading (β = −0.15; 95% CI −0.20 – −0.09; p<0.001) and writing ability (β = −0.14; 95% CI −0.19 – −0.09; p<0.001) for each one CBCL-att z-score increase, when compared to those in the morning shift, which presented a smaller decrease for reading (β = −0.07; 95% CI −0.12 – −0.02; p=0.006) and writing ability (β = −0.07; 95% CI −0.11 – −0.02; p=0.007) for one CBCL-att z-score increase (Supplementary Table A3). Figures 1E and 1F depict a more pronounced decline in reading and writing abilities among students attending the afternoon shift compared to those in the morning shift, conditioned on inattention/hyperactivity problems assessed by CBCL.

Three years later, higher CBCL-att at baseline was associated with worse performance in school subjects and higher chance for negative school events, independently from covariates and baseline levels of these outcomes, replicating previous findings with ADHD and SDQ (Supplementary Table A9). Morning shift at baseline also predicted higher chance for negative school events, replicating findings using SDQ-hyper in the regression models (Supplementary Table S9).

## Discussion

The present study examine the impact of the school shift on children and adolescents with ADHD and the educational consequences of this interplay, cross-sectionally and longitudinally. Cross-sectional analysis was design to understand if those with ADHD and attending classes in the morning were also struggling with educational problems. On the other hand, longitudinal analysis estimated the ADHD and school shift associations with the outcomes independently from the baseline levels of the educational outcomes. Confirming our hypothesis, we found that those with ADHD studying in the morning shift presented lower performance in academic subjects when compared with those with ADHD in the afternoon shift and those without ADHD. However, this finding was not replicated in sensitivity analysis. Moreover, contrary to our hypothesis, the most consistent finding was that those studying in the morning, regardless on the ADHD status, already performed worse in reading and writing ability, as well as they present higher odds for the composite of suspension, retention, and dropout. Thus, the interaction found was in the sense that those studying in the afternoon have room to decrease their performance on reading and writing ability as ADHD symptoms increase. While investigating if these associations remain thorough time, we found independent negative associations between ADHD and school shift with educational outcomes. Nonetheless, and contrary to our hypothesis, these interactive associations are cross-sectional only and were not found in the longitudinal analysis.

The impact stemming from the independent associations of ADHD and school shift are important to contextualize the study’s findings. Young people with ADHD presented lower levels of performance in school subjects and higher odds for negative school events and these associations remain three years later, independently from their prior levels of these educational outcomes. At the cross-sectional level only, ADHD is also associated with lower reading and writing ability. These associations are consistent with previous literature demonstrating the impact of ADHD in educational outcomes [1, 2, 18, 34]. We add value to previous findings by demonstrating that this is independent from developmental and educational stage, social class, race/ethnicity and IQ and not sensitive to operationalization of ADHD, if at the diagnostic or the dimensional level.

In a similar way, previous evidence demonstrate that morning school shift is associate with lower academic performance in several school subjects, including reading and writing ability [8, 10–13]. Chronotype might explain this association. At this age, children and adolescents have a later chronotype (i.e., tendency to prefer doing activities in the evening or night) and morning shifts are not in synchrony with their academic activities during the morning schedule [10, 11, 35]. Moreover, selecting the school shift based on individual chronotype might reduce negative school events such as grade retention [10, 11, 35]. Our study, however, has not accessed chronotype, and mechanisms remain to be further investigated.

Our findings challenge the notion, and our hypothesis, that individuals with ADHD present worse academic performance when attending morning classes. Instead, we found that the ADHD sets a limit for educational outcomes, meaning that morning shift no longer add to worsen these outcomes when this condition is set (i.e., it keeps only its independent association). In a different perspective, ADHD increases the chances for worse educational outcomes only if children and adolescents attend the afternoon shift, as the morning shift already impairs educational performance, also setting a limit for it. It is important to highlight that the observed interaction is confined to cross-sectional analyses, suggesting that this can be a risk marker or, if causal, these interactions fade away with time. We cannot dismiss the possibility that some participants changed their school shift between baseline and follow-up, potentially lessening the educational impairments linked to the baseline shift. However, given that parents did not have the option to choose the school shift, it is improbable that any shift changes would have diminished the baseline impact on educational measures at follow-up.

Furthermore, interactions were sensitive to ADHD operationalization (diagnostic vs dimensions) when compared with the independent associations. However, it was not sensitive between dimensional instruments. This underscores the importance of scrutinizing the impact of methodological choices and reinforces the problems of current nosologically systems to detect those at risk of poor outcomes due to psychopathology. Regarding methodological choices, dichotomizing variables can lead to oversimplification, potentially obscuring meaningful associations due to loss of statistical power and weakening the study’s ability to detect subtle but significant associations [36]. Regarding nosology, there is strong evidence that ADHD is composed by dimensions of psychopathology that are continuously distributed [21, 37–39]. Moreover, previous evidences suggests that dimensions of psychopathology have advantages over categorization, as it explain more variance of several outcomes associated with mental health problems [40–43]. Thus, dimensional approaches are relevant to be used in analysis dealing with inherently continuous constructs. The sensitivity analysis employed in this study shed some light on the relationship between school shift and ADHD with educational outcomes by demonstrating that dimensional measures of ADHD can be more useful to detect those at risk for poor reading and writing ability, especially if studying in the afternoon shift.

The study has strengths as well as limitations. As strengths, the study focuses on a sample of students in a large LAMIC, where double shift was not chosen by students’ parents. Furthermore, we used adjustments to control for attrition, representativeness, and confounding variables, such as IQ, and the utilization of standardized tests for outcome measures bolster the internal validity of the study. Additionally, the employment of dimensional measures for ADHD allows for a nuanced understanding of the results, revealing robustness to the chosen measurement approach. The first limitation is the inherent nature of observational design hampers causality claims. We minimized reverse causality by adjusting the longitudinal analysis for confounding factors and for baseline levels of the outcomes to examine the additional effect of the adjusted predictors. Nonetheless, the analysis demonstrate that ADHD and school shift are at least markers of poor educational outcomes at least. Second, there is the possibility that parents might alter their child’s school shift in response to emerging academic difficulties. However, this scenario appears unlikely in public schools where parental choice is limited. Third, the absence of information regarding the participants’ chronotype in our study represents a notable limitation, as the interaction between ADHD, school timing, and the individual’s preference for morning or evening activities remains unexplored. However, chronotype is beyond the scope of the present study and future studies should examine its interaction with present findings. Fourth, constraint lies in the study’s sample characteristics, with an average age of 10 years and a follow-up period of 3 years, potentially limiting the generalizability of the findings to younger children or older adolescents. Fifth, the sample comprises students from two Brazilian capital cities, primarily high-risk for a familial history of psychopathology, raising concerns about external validity across different countries and necessitating replication studies in diverse populations. Nonetheless, we minimized that by adjusting the analysis for study site, used weights to account for the oversampling procedure and for follow-up attrition.

## Conclusion

This study examined the interplay between ADHD and school shift with educational outcomes. Our results confirm the hypothesis that ADHD and morning shift are factors associated with poor educational outcomes. However, interactions were found only at the cross-sectional level and in an unexpected direction. We found that those with low levels of ADHD symptoms have better performance in reading and writing tests if they are attending afternoon classes. Our findings support that one possible explanation for this phenomenon is that the morning school shift sets a bottom limit for educational outcomes and, therefore, does not worsen the association between ADHD and educational outcomes. As we found only cross-sectional interactions in this observational study, it is possible that this association is only marking a risk for poor outcomes. Thus, experimental studies are needed to clarify whether children and adolescents with ADHD would benefit from studying in a school shift that respects their chronotype and rhythm of the circadian rhythm.

## Data Availability

Data dictionary is available at https://osf.io/ktz5h/wiki/Data%20Dictionaries/and https://osf.io/w3jr4 to direct download. Individual-level data is available upon request to the Brazilian High-Risk Cohort Study research committee, by following the instructions and filling the research form available at https://osf.io/ktz5h/wiki/home/. Study design and ethical details can be found elsewhere [14].
